# Wide-Bandgap Halide Perovskites for Indoor Photovoltaics

**DOI:** 10.3389/fchem.2021.632021

**Published:** 2021-03-26

**Authors:** Lethy Krishnan Jagadamma, Shaoyang Wang

**Affiliations:** Energy Harvesting Research Group, SUPA, School of Physics and Astronomy, St. Andrews, Scotland, United Kingdom

**Keywords:** composition tuning, triple cation, triple anion, CH_3_NH_3_PbI_3_, internet of things, power conversion efficiency, indoor light spectra

## Abstract

Indoor photovoltaics (IPVs) are receiving great research attention recently due to their projected application in the huge technology field of Internet of Things (IoT). Among the various existing photovoltaic technologies such as silicon, Cadmium Telluride (CdTe), Copper Indium Gallium Selenide (CIGS), organic photovoltaics, and halide perovskites, the latter are identified as the most promising for indoor light harvesting. This suitability is mainly due to its composition tuning adaptability to engineer the bandgap to match the indoor light spectrum and exceptional optoelectronic properties. Here, in this review, we are summarizing the state-of-the-art research efforts on halide perovskite-based indoor photovoltaics, the effect of composition tuning, and the selection of various functional layer and device architecture onto their power conversion efficiency. We also highlight some of the challenges to be addressed before these halide perovskite IPVs are commercialized.

## Introduction

**FIGURE 1:**
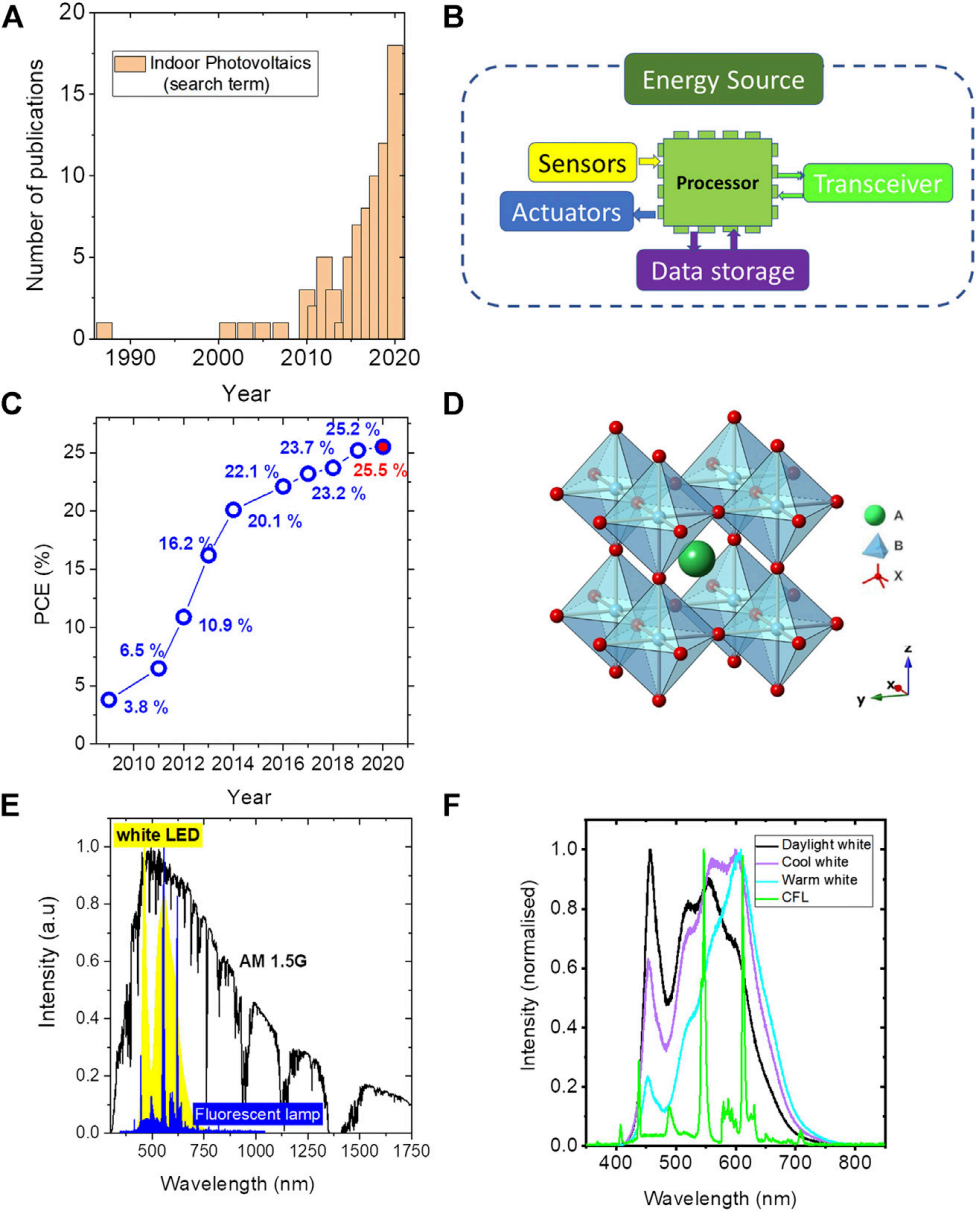
**(A)** The graph showing the number of IPV related publications as a function of year. **(B)** The main components in an IoT system. **(C)** The advancement of PCE for hybrid perovskite solar cells over the last decade. **(D)** Perovskite crystal structure with cubic symmetry. Organic or inorganic cations occupy position A (green) whereas metal cations and halogens occupy the B (bluish-grey) and X (red) positions, respectively. **(E)** Comparison of 1 Sun spectrum to indoor artificial light spectra. **(F)** Spectra of different types of white LEDs and CFL lamp used inside the buildings.

Photovoltaic devices convert light to electricity. The term has already become synonymous with solar cells, which are a type of photovoltaic device in which the incident light is sunlight. There are indoor photovoltaic (IPV) devices that convert light from artificial light sources such as white light-emitting diode (LED) and fluorescent lamps inside the buildings to electrical energy (Chen, 2019; Li et al., 2020). The history of indoor photovoltaics dates back to the 1970s when the amorphous silicon solar cells were used to power watches and calculators (Minnaert and Veelaert, 2014). For over 40 years, silicon solar cells were the ones mainly used for harvesting indoor light, and IPV research field was almost stagnant until 2010. However, over the last five years, IPVs are gaining much research attention and this is reflected in the increasing number of research publications on this topic published every year (Figure 1A). This increasing interest can be attributed to the combined effect of four main factors: 1) emergence of efficient third-generation thin-film solar cells such as organic and hybrid perovskites; 2) replacement of incandescent lights inside the buildings by solid-state white LEDs and fluorescent lamps (FLs); 3) the boom of disruptive technology such as the Internet of Things (IoT) and the associated unprecedented commercial opportunities; and 4) the continued decrease of power requirement for wireless sensors.

The concept of the IoT, initially coined by Kevin Ashton in 1999, has now projected to be a 1.6 trillion market in 2025 (Kramp et al., 2013). IoT is a smart network of connected physical objects with embedded sensors and actuators. IoT industry is projected to make an economic impact of $11 trillion by 2025 and as many as 75 billion connected IoT devices. Furthermore, it is noticeable that half of those components to be installed will be located inside buildings (Mathews et al., 2019). Wireless sensors are the most fundamental components in these smart devices (Figure 1B) (Davies, 2015). Sustainably, powering these sensors is a huge challenge. At present, these sensors are powered by batteries which limit the IoT potential by service interruptions due to battery replacement and eventually cause an environmental problem (due to battery disposal). These sensors only require µW-mW of power for their efficient functioning. Light energy is available in the ambient environment and can be accessed easily via photovoltaic devices without requesting additional devices or multiple energy transfer, thus becoming the most promising candidate to power IoT sensor system.

Different types of PV materials including III-IV, CIGS, organic, dye-sensitized, and perovskite are taken into consideration for efficient indoor light harvesting. Table 1 shows the main photovoltaic materials available and their bandgap. In contrast to conventional solar cell technologies such as silicon, the emerging PV technologies based on organic and halide perovskite semiconductors have tunable bandgap. In the history of photovoltaics, no other light harvesting material has ever triggered research attention and promising avenues to harness solar energy similar to organometal halide perovskites (Jena et al., 2019; Miyasaka et al., 2020; Mhaisalkar et al., 2020). Within a decade, the power conversion efficiency (PCE) of these perovskite solar cells has made an amazing advancement from 3.81% in 2009 to 25.5% today (Figure 1C). Represented by a general chemical formula of ABX_3_, this family of materials provide a framework to bind organic and inorganic component to a molecular composite, where A is an organic or inorganic cation, B is a divalent cation, such as Pb^2+^ and Sn^2+^, and X is halogen, I^−^, Br^−^, or Cl^−^. Organic cations, A, can be methylammonium (MA) CH_3_NH_3_^+^, ethylammonium (EA) C_2_H_5_NH_3_^+^, formamidinium (FA) HC(NH_2_)_2_^+^, and even inorganic Cs (Figure 1D). These materials possess exceptional optoelectronic properties needed for a photovoltaic material such as direct bandgap which is tunable (1.17–3.3 eV), high absorption (absorption length of 200–300 nm) in the visible range, low exciton binding energy ∼30 meV (comparable to RT thermal energy), high electron and hole bulk mobility (∼2000 cm^2^/(V.s) and ∼300 cm^2^/(V.s), respectively), large diffusion length (>1 μm), and ability to form high-quality crystals at relatively low processing temperatures (RT to 150°C) via vacuum or wet synthesis methods (Grätzel, 2014; Stoumpos and Kanatzidis, 2015; Green et al., 2015; Park, 2015).

**TABLE 1:** Different photovoltaic materials and their bandgap.

Photovoltaic material	Bandgap (eV)	Tunable (bandgap)
Crystalline silicon (c-si)	1.1	X
Amorphous silicon (a-Si)	1.7	X
CdTe	1.45	X
CIGS	1.01–1.68	✓
Organic photovoltaics	1.0–2	✓
Halide perovskites	1.1–3.3	✓

The distributed nature of the IoT sensors requires that the indoor light energy harvesters also be distributed in nature. Thus, IPVs, which are suitable for powering the sensors, need to have high PCE, ease of processability, low cost, earth abundance of constituting materials, flexibility, conformability, and lightweight. Halide perovskite photovoltaic devices integrate all these requirements. Along with the extraordinary power conversion efficiency under one sun, halide perovskites have shown remarkable efficiency under indoor lighting as well. Even though recently Li et al. (2020), Chen (2019), and Lee et al. (2019b) have reviewed thin-film indoor photovoltaics, the growing potential and increasing number of publications related to halide perovskite IPVs demand a review focusing on the halide perovskite IPV itself.

### Factors Affecting Indoor Light Harvesting

The common perception is that high-performance Si solar cells can perform the same under indoor lighting condition. This is not true and is especially pertinent with the phasing out of the incandescent light bulbs which have a similar broad emission spectrum to that of the solar spectrum. Identifying which photovoltaic technology is suitable for IPV depends mainly on the 1) indoor light spectrum and 2) factors determining the power conversion efficiency.

### Indoor Light Spectrum and Maximum Theoretical Efficiency of IPVs

Indoor lighting in residential buildings and offices is now dominated by fluorescent lamps and white light-emitting diodes which are entirely different in intensity (1,000 times lower) and spectral content (narrow spectrum vs. broad) from the solar spectrum (Figure 1E). The intensity of standard sunlight spectrum (AM 1.5G) is 100 mW/cm^2^ and the typical indoor artificial light intensity is 0.05–1 mW/cm^2^ (∼200–2000 lux). The low available light intensity inside the building was one reason why indoor photovoltaic technology was not receiving much attention for many years. However, when the IoT gained popularity since 2010, indoor photovoltaics also started receiving its attention. This is because the IoT sensors only require µW-mW of power to operate. Depending on the correlated color temperature, three main types of white LED lighting used inside buildings are warm white, cool white, and daylight white. The typical spectra of these light sources are shown in (Figure 1F). As shown in the spectra, a warm white LED has a higher orange spectral range compared to blue; cool white LED has higher blue spectral content compared to warm white. Inside buildings, warm white LEDs are usually used in living rooms, bedrooms, and hallways. Cool white LEDs are usually used in kitchens, study areas, bathrooms, offices, and retail stores; daylight LEDs are used in commercial, retail, and art studios (integral-led.com).

**FIGURE 2:**
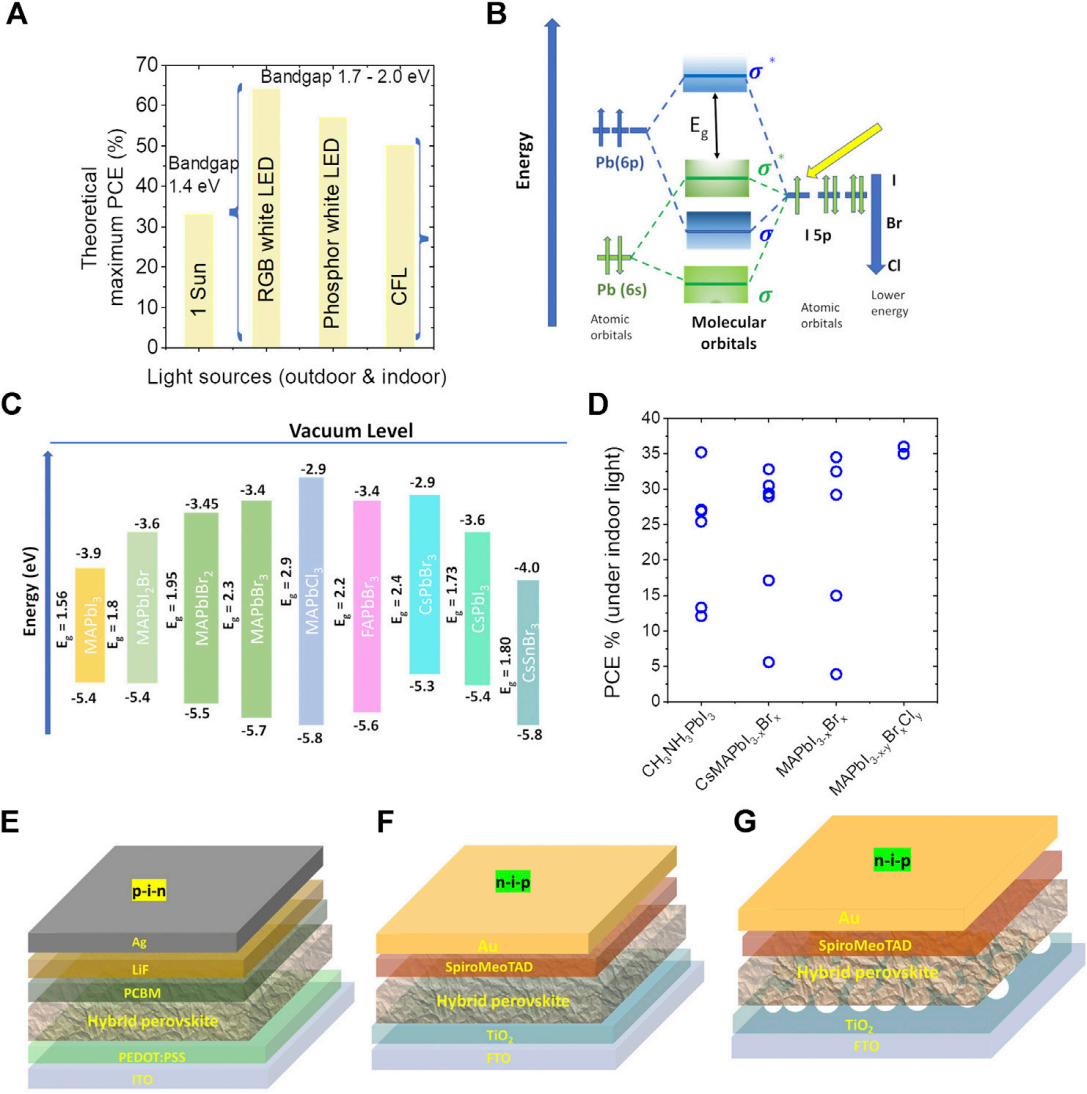
**(A)** Maximum theoretical efficiency of IPVs as a function of bandgap, **(B)** electronic band structure of halide perovskites, **(C)** some typical wide-bandgap halide perovskites, and **(D)** power conversion efficiency of halide perovskite indoor photovoltaic devices as a function of different composition. Different photovoltaic device architectures, **(E)** planar *p-i-n*, **(F)** planar *n-i-p*, and **(G)** mesoporous *n-i-p* architectures.

Different spectral content and wavelength range of indoor light sources mean that the optimum bandgap and thermodynamically limited maximum power conversion efficiency (Shockley-Queisser limit) are different for indoor photovoltaics compared to outdoor solar cells. Under the solar spectrum (100 mW/cm^2^), the maximum theoretical efficiency is 33% and the optimum bandgap is 1.4 eV (Shockley and Queisser, 1961). In the case of indoor lighting conditions, the optimum material bandgap is ∼1.9 eV and the maximum theoretical efficiency can reach as high as ∼60% for LEDs and 50% for fluorescent lamps (Müller et al., 2013; Ho et al., 2020) (Figure 2A). This implies that the active layer composition of the perovskite indoor photovoltaics needs to be modified for higher photon energy indoor lightings compared to that of 1 Sun. This aspect is further detailed in *Band Structure of Halide Perovskite Semiconductors*.

### Indoor Photovoltaic Performance Parameters

The photovoltaic power conversion efficiency (ƞ) is determined by three performance parameters open-circuit voltage (Voc), short circuit current density (Jsc), and fill factor (FF) as

$$\eta \left(\%\right)=\frac{J_{sc}\times \;V_{oc}\times FF\times 100\;\%}{P_{\text{in}}},$$

where $P_{in}$ is the input light intensity. The dependence of these performance parameters on the incident light intensity is shown as follows (Goo et al., 2018; Koster et al., 2005):

$$J_{sc}\;\infty \;I^{\alpha },$$ (1)

$$V_{oc}=\frac{nk_{B}T}{q}\ln\left(\frac{J_{sc\;}}{J_{o}}+1\right),$$ (2)

where *I* is the incident light intensity, α is the recombination factor n is the ideality factor, $k_{B}$ is the Boltzmann factor, and $J_{o}$ is the diode saturation current/leakage current.

FF depends indirectly on the light intensity through the following relation (Green, 1981):

$$FF_{s+sh}=FF_{s}\left[1-\frac{\left(v_{oc}+0.7\right)}{v_{oc}}\;\frac{FF_{s}}{r_{sh}}\right],$$ (3)

$$FF_{s}=\;FF_{0}\left(1-r_{s}\right),$$ (4)

where $FF_{s+sh}$ is the FF taking into account both series and shunt resistance, $FF_{s}$ is the FF considering only the series resistance,$\left(\;R_{s}\right)$ and $FF_{0}\;$ is the ideal FF without taking into consideration either series or shunt resistance $\left(R_{SH}\right)$.

$v_{oc}$ is the normalized open-circuit voltage given by

$$v_{oc}=\;\frac{V_{oc}}{V_{T}},$$ (5)

where $V_{T}$ is the thermal voltage,

$$V_{T}=\frac{k_{B\;}T}{q}\;,$$ (6)

where $r_{s}$ and $r_{SH}$ are the normalized series and shunt resistance given by

$$r_{s}=\frac{R_{s}}{R_{CH}},$$ (7)

$$r_{SH}=\frac{R_{SH}}{R_{CH}},$$ (8)

where $R_{CH}$ is the characteristic resistance, expressed as

$$R_{CH}=\frac{V_{OC\;}}{\left(J_{SC}\times A\right)},$$ (9)

where A is the area of the photovoltaic device. Under the low light intensity, $J_{sc}$ falls faster than $V_{oc}\;\;$ due to its power-law dependence. Also, the influence of shunt resistance on FF becomes high (Eq. 3) and the dependence on Rs is relaxed, which is in direct contrast with the FF dependence of $R_{SH}$ and $R_{s}$ under 1 Sun (Steim et al., 2011). This requires stringent interface engineering to minimize the recombination losses of the photogenerated carriers.

In this review, we will be mainly focusing on the wide-bandgap halide perovskite semiconductors suitable for indoor light harvesting. Halide perovskite bandgap can be tuned from 1.1 to 3.7 eV by interchanging/mixing B cation (Ge, Sn, and Pb) or halogens (X = Cl, Br and I) (Hu et al., 2019; Jena et al., 2019; Mhaisalkar et al., 2020). This is highly advantageous considering the different spectral features of the indoor light sources. Since the bandgap modification is required for maximizing indoor light harvesting, understanding the band structure of halide perovskite is highly important and constitutes the focus of discussion for the next section.

### Band Structure of Halide Perovskite Semiconductors

Electronic band structure determines the optoelectronic properties such as optical absorption of the incoming photons, charge transfer from the active layer to the charge-transporting layers, and opportunity for bandgap engineering. Halide perovskites are direct bandgap semiconductors with sharp absorption edges. In halide perovskites, both the valence band maximum (VBM) and conduction band minimum (CBM) are constituted by antibonding sigma orbitals but with different degree of contribution from B cation and X anion s and p orbitals (Walsh, 2015; He et al., 2017). The A cation has no direct contribution to the electronic band structure but has a strong indirect influence via the octahedral tilting and hence the B-X-B bond angles (Kang and Wang, 2017; Elumalai et al., 2016; Stoumpos and Kanatzidis, 2015). Through steric and Coulombic interactions, A cations deform the BX_6_ octahedral unit, and this tilting changes the absorption edges and electronic bandgap. The valence band is formed by mixing of halide np ^6^ orbitals (where n is the principal quantum number) and metal (B cation) *ns* ^2^ orbitals with a major contribution from halide *np* ^6^ orbitals (and a minor contribution from the ns ^2^). The conduction band is constituted by mixing of B cation’s np orbital with halide’s np ^6^ orbital with a major contribution from metal p orbital (and a minor contribution from halide p orbital).

As the halide ions are moved from I (5p ^6^) to Br (4p ^6^) to Cl (3p ^6^), the energies of the halide np ^6^ orbitals downshift the VBM by 0.8 eV and shift the CBM by 0.19 eV, thus widening the bandgap (Stoumpos and Kanatzidis, 2015; Ravi et al., 2016; Walsh, 2015; Olthof, 2016; He et al., 2017). However, replacing the Pb with Sn, the conduction band is downshifted with Sn 5p and reduces the bandgap. It is worth noting that the wide-bandgap perovskites are important for Si/perovskite (1.7 eV) and perovskite/perovskite (1.8 eV) tandem solar cells (Tong et al., 2020). When selecting the suitable wide-bandgap perovskites for indoor solar cells, the bandgap of the bottom cell perovskite composition can be used as a good starting point for optimizing the bandgap required for indoor photovoltaics. Taking CH_3_NH_3_PbI_3_ as a typical example, the band structure of halide perovskite is shown in Figure 2B (He et al., 2017; Huang et al., 2017). Figure 2C shows the energy level structure of some wide-bandgap halide perovskites (Tao et al., 2019; Wu et al., 2018; Huang et al., 2019).

With this background understanding of band structure of halide perovskites, in the next section, the indoor photovoltaic properties of halide perovskites would be reviewed with special emphasis on the selection of the active layer materials and the device architecture.

### Halide Perovskite-Based Indoor Photovoltaics

Most of the initial reports on the indoor photovoltaic performance of hybrid perovskites were focused on the most widely investigated hybrid perovskite composition of CH_3_NH_3_PbI_3_. CH_3_NH_3_PbI_3_ has a bandgap of 1.56 eV, lower than the theoretically predicted optimum bandgap reported for indoor photovoltaics. By interface engineering and controlling the traps at the interfaces and carrier dynamics, CH_3_NH_3_PbI_3_-based indoor photovoltaic devices have demonstrated a power conversion efficiency ranging from 20% to 34% under indoor lighting conditions (Chen et al., 2015; Dagar et al., 2018; Lucarelli et al., 2017; Di Giacomo et al., 2016). Raifuku et al. (2016) have previously shown that hybrid perovskite solar cells perform better under low-intensity illumination such as 0.1 mW/cm^2^, compared to crystalline silicon. They demonstrated that, at low-intensity levels, perovskite solar cells can retain 70% of their open-circuit voltage at 1 Sun. Concerning interface engineering in indoor photovoltaics, the study reported by Li et al. is highly significant as they demonstrated that the ionic liquid of 1-butyl-3-methylimidazolium tetrafluoroborate ([BMIM]BF4) can passivate the surface traps on the electron transport layer of PCBM and prevent moisture and oxygen erosion to the perovskite active layer. These indoor photovoltaic devices based on CH_3_NH_3_PbI_3_ showed a power conversion efficiency of 35.2% under indoor lighting conditions (Li et al., 2018).

We have previously reported that, compared to CH_3_NH_3_PbI_3_, the mixed halides of iodide-bromide and iodide-chloride can have a higher power conversion efficiency of 23% under indoor white LED and CFL lighting (Jagadamma et al., 2019). In 2018, Guo et al. showed that CH_3_NH_3_PbI_3_ doped with Cl and citric acid can convert indoor white LED light to electricity with 26% power conversion efficiency. They attribute this enhanced efficiency to the perovskite crystal modulation effects and their improved quality (Guo et al., 2017). Very recently, Sun et al. reported bandgap engineered Cs_0.05_MA_0.95_PbBr_x_I_3-x_ perovskites with a systematic variation of its bandgap from 1.6 to 1.75 eV by increasing the bromide to iodide ratio. These solar cells demonstrated a record efficiency of 36% under white light-emitting diode and 33.2% under CFL lamp (Sun et al., 2020). Using the similar approach of composition engineering to tune the bandgap of CH_3_NH_3_PbI_3_, that is by increasing the bromide to iodide ratio (1.56 eV–1.76 eV), very recently Lim et al. have also reported a power conversion efficiency of 34% under white LED illumination (Lim et al., 2020). Triple cation and triple anion-based perovskite solar cells have also been tested for their efficiency under indoor lighting conditions. Under white LED illumination, CsFAMA triple cation-based devices demonstrated a PCE of 21% (Mica et al., 2020). By adjusting the anion I/Br/Cl content in the MAPbI_3−x-y_Br_y_Cl_x_ composition, the bandgap was tuned from 1.6 to 1.8 eV and record indoor PCE of 36% was demonstrated (Cheng et al., 2019). It should be noted that, among the various perovskite-based IPVs reported so far, this bandgap-tuned composition of 1.8 eV has resulted in the highest Voc (>1 V) under 1,000 lux white LED indoor illumination. Pb-free perovskites are also gaining attention in indoor PV application and a very recent report showed a PCE of 4.5% under white LED illumination (Peng et al., 2020). In Figure 2D, the power conversion efficiency of halide perovskite indoor photovoltaics as a function of their composition is provided. Figure 2D shows that the CH_3_NH_3_PbI_3_ solar cells with interface engineering are giving a comparable performance to bandgap engineered wide-bandgap semiconductors. This implies that there is enough room to improve the PCE of wide-bandgap halide perovskites by interface engineering and modifying the device architecture. To understand the role of device architecture on the Voc loss (and hence on power conversion efficiency), indoor photovoltaic efficiency as a function of different architecture is reviewed in the next section.

This is partly motivated by the report by Raifuku et al., where they showed that, under low light intensity, planar, structure outperforms mesoporous architecture and retains better Voc under low-intensity illumination (Raifuku et al., 2016). The two common device architectures in perovskite solar cells are *n-i-p* and *p-i-n* as shown in Figures 2E and 2F. In *n-i-p* configuration, there is mesoporous and planar device configuration. Table 2 lists a summary of PCE (and Voc) of halide perovskite indoor photovoltaics as a function of device architecture. Both *n-i-p* and *p-i-n* PV device architectures are found to be showing good power conversion efficiencies under indoor lighting. Previously, Lee et al. have reported the significance of selecting different device architectures and the functional layers in maximizing the power output of indoor photovoltaics devices (Lee et al., 2019a). They have noticed that in mesoporous *n-i-p* architecture replacing the Spiro-MeOTAD hole transporting layer with PTAA reduces the output power dramatically and in inverted *p-i-n* architecture, replacing the PEDOT:PSS HTL by Poly-TPD enhances the maximum output power.

**TABLE 2:** PCE and V_oc_ of some typical halide perovskite-based indoor photovoltaics with different functional layers and device architecture.

Device configuration	Architecture and functional layers	Voc (V) (indoor)	PCE (indoor)
p-i-n	ITO/PEDOT:PSS/CA- MAPbI_3_/PCBM/PEIE/Ag Guo et al. (2017)	0.81	28.1
p-i-n	ITO/NiOx/MAPbI_3_/PCBM/BMIMBF4/PCBM/Ag Li et al. (2018)	0.87	35.2
p-i-n	ITO/NiOx/MAPbCl_0.1_I_2.9_/PCBM/LiF/Ag Jagadamma et al. (2019)	0.90	23
n-i-p planar	ITO/NbO_x_-TiO_2_/CsMAPbIBr/Spiro/Au Sun et al. (2020) higher incident input power (0.8 mW/cm^2^)	0.999	36.3
p-i-n	ITO/NiO/MAPbI_2-x_BrCl_x_/PCBM/BCP/Ag Cheng et al. (2019)	1.03	36.2
n-i-p planar	ITO/TiO_2_/MAPb_3-X_Br_x_/Spiro/Au Lim et al. (2020)	0.82	34.5
n-i-p (meso)	FTO/TiO_2_/m-TiO2/MAPI/Spiro/Au Di Giacomo et al. (2016)	0.350	4.8 (10.6 under one sun)

## Challenges and Future Outlook

Halide perovskite semiconductors have thus already demonstrated their remarkable potential in realizing highly efficient IPVs. However, along with further efforts toward achieving the theoretically predicted PCE and accelerating their real-life application in IoT systems, the following challenges need to be addressed.

### Photo-Induced Phase Segregation

Normally, wide-bandgap halide perovskites are obtained by replacing iodide (I-) ions with bromide (Br-), to form mixed halides. However, when the Br content is more than 20%, halide phase segregation (iodide rich and bromide rich phases) occurs upon photoexcitation of mixed halide perovskites (Hoke et al., 2015). The iodide-rich regions act as traps for photogenerated carriers and limit the open-circuit voltage in the corresponding solar cells. So, the advantage of the wide bandgap is not reflected in the open-circuit voltage of the IPV as expected (Yang et al., 2018). The voltage deficit can be expressed as $V_{deficit}=\;\frac{E_{g}}{q}-V_{oc}.$ The additive strategy and 3D/2D heterostructure are found to be promising to suppress the phase segregation effects and hence to overcome the $V_{oc}$ loss (Tong et al., 2020).

### Stability and Pb Toxicity

Like outdoor perovskite solar cells, the indoor perovskite solar cells should also demonstrate high stability and robust encapsulation to prevent any Pb leakage to the indoor ambient. Compared to outdoor conditions, inside the buildings, these devices are exposed to mild weather conditions in terms of temperature and humidity. Even though extensive stability characterization standards are available for outdoor solar cells, no standard stability test conditions exist for indoor photovoltaics and a new stability standard has to be developed for indoor photovoltaics (Khenkin et al., 2020).

### S-Shape Challenge

In indoor photovoltaic devices using metal oxide transport layers such as SnO_2_, TiO_2_, ZnO, there is a possibility that these devices suffer s-shape challenge (light soaking effect) limiting the power conversion efficiency (Jiang et al., 2018; Yan et al., 2017). These metal oxide layers have defects which are filled during the 1 Sun measurement due to the UV-spectral component and high incident light intensity. However, since indoor light has no UV-spectral component and low intensity, the s-shape challenge is more critical for indoor photovoltaics. More research efforts would need to develop metal oxide charge-transporting layers suitable for indoor applications and need no UV excitation to fill the trap states.

### V_oc_ Loss

Voltage loss of the indoor perovskite photovoltaic devices is much higher compared to the outdoor 1 Sun solar cell devices. Comparing the optimum bandgap differences (1.4 eV vs. 1.9 eV), presently, the Voc loss is more than 1 V for the indoor PVs even if the intensity differences are accounted. One main reason is that wide-bandgap perovskite solar cells usually suffer from high Voc losses with respect to their theoretical limit (Mahesh et al., 2020). There should be more research efforts to enhance the Voc improvement of indoor perovskite solar cells to achieve the theoretically predicted maximum power conversion efficiency of 50%–60%.
